# LncRNA BMNCR Regulates Proliferation, Apoptosis and Inflammatory Response in Bovine Mammary Epithelial Cells Through the miR-145/ANO6 Axis

**DOI:** 10.3390/ani16101446

**Published:** 2026-05-08

**Authors:** Tianqi Zhao, Xubin Lu, Shuangfeng Chu, Yadan Chen, Jiayi Zhou, Fengqi Zhao, Yujia Sun, Zhangping Yang

**Affiliations:** 1 College of Animal Science and Technology, Yangzhou University, Yangzhou 225009, China; dx120220167@stu.yzu.edu.cn (T.Z.); lxb@yzu.edu.cn (X.L.); shfchu@163.com (S.C.); ydanchen23@163.com (Y.C.); 15189462492@163.com (J.Z.); 2 Joint International Research Laboratory of Agriculture & Agri-Product Safety, Ministry of Education, Yangzhou University, Yangzhou 225009, China; 3 Zhejiang Key Laboratory of Cow Genetic Improvement and Milk Quality Research, Wenzhou 325000, China; 4 Department of Animal and Veterinary Sciences, University of Vermont, Burlington, VT 05405-0148, USA; fzhao@uvm.edu

**Keywords:** bovine mastitis, *S. aureus*, lncRNA BMNCR, bta-miR-145, *ANO6*

## Abstract

Bovine mastitis causes significant economic losses in the dairy industry. Emerging evidence highlights the critical role of long non-coding RNAs (lncRNAs) in inflammation-associated epigenetic regulation through competing endogenous RNA (ceRNA) networks. In this study, we established a bovine mastitis model in three healthy primiparous Holstein cows by intramammary infection with *S. aureus*. Infected and control mammary tissue samples were then collected for transcriptomic profiling, which identified 2005 differentially expressed lncRNAs. Among them, BMNCR was significantly upregulated in *S. aureus*-infected mammary tissues and *S. aureus*-stimulated BMECs. We evaluated the coding potential of BMNCR and confirmed its non-coding nature. Functional studies in BMECs demonstrated that knockdown of BMNCR suppressed proliferation, promoted apoptosis, and altered the expression of inflammatory factors, including IL-2, IL-6, IL-8, and IL-12. Mechanistically, BMNCR acted as a sponge for bta-miR-145, thereby leading to the derepression of *ANO6*. Silencing *ANO6* partly recapitulated the effects of BMNCR knockdown, impairing proliferation and increasing IL-8 expression. Collectively, these findings suggest that the BMNCR/miR-145/*ANO6* axis is involved in the regulation of inflammatory responses and epithelial homeostasis during bovine mastitis, with BMNCR functioning as a protective regulator in this process.

## Simple Summary

Bovine mastitis could affect cow health, lower milk quality, and result in substantial economic losses in dairy production. In this study, we identified and designated bovine mastitis-related long non-coding RNA (BMNCR) as BMNCR, which was markedly upregulated in *Staphylococcus aureus* (*S. aureus*)-infected mammary tissues and *S. aureus*-stimulated bovine mammary epithelial cells (BMECs). We found that reducing BMNCR suppressed BMEC proliferation, promoted apoptosis, and altered inflammatory cytokine expression. Further analysis showed that BMNCR may affect mastitis by regulating the interaction between bta-miR-145 (miR-145) and Anoctamin 6 (*ANO6*). These findings improve our understanding of bovine mastitis and suggest that BMNCR may function as a protective regulator that helps maintain mammary epithelial cell homeostasis.

## 1. Introduction

Bovine mastitis represents one of the most prevalent diseases in dairy cattle, incurring substantial economic losses to the global dairy industry [1]. Bovine mastitis is categorized into clinical and subclinical types: clinical mastitis manifests visible abnormalities in milk secretion (flakes or clots) accompanied by udder inflammation, while subclinical mastitis is characterized by elevated somatic cell counts (SCC) in milk, primarily comprising macrophages, polymorphonuclear leukocytes, lymphocytes, and epithelial cells [2]. Notably, subclinical mastitis is more prevalent than clinical mastitis and frequently escapes early detection due to the absence of overt symptoms [3]. *S. aureus*, a predominant etiological agent in bovine mastitis cases, frequently escapes host immune surveillance and establishes persistent infections, especially in subclinical cases [4]. Traditional management strategies, including antibiotic treatment and improved milking hygiene, demonstrate limited efficacy in controlling *S. aureus*-associated mastitis [5]. Furthermore, prolonged antibiotic usage not only promotes antimicrobial resistance but also raises public health concerns regarding potential drug residues in dairy products [6].

Recent advancements in molecular genetics have introduced new avenues for understanding and combating bovine mastitis [7]. Among these, long non-coding RNAs have garnered attention as crucial epigenetic regulators [8,9]. Defined as non-protein-coding RNA molecules exceeding 200 nucleotides in length, lncRNAs exert regulatory effects on cellular and developmental processes through diverse interactions with other biological molecules such as transcription factors, miRNAs, RNA-binding proteins and chromatin [10,11]. Notably, lncRNAs can function as competing endogenous RNAs, competing with miRNAs for binding to target mRNAs or sequestering miRNAs by occupying their shared binding sites, thereby influencing gene expression [12]. This lncRNA/miRNA/mRNA interaction network, referred to as the competing endogenous RNA (ceRNA) network, has been implicated in various pathological processes, including mammary-related diseases [13,14]. For instance, lncRNA CA12-AS1 promotes the expression and secretion of inflammatory factors and apoptosis by targeting miR-133a, inhibits cell proliferation, thereby aggravating the inflammatory response of LPS-induced BMECs [15]. Similarly, lncRNA TCL6 acts as a ceRNA for miR-876-5p, attenuating its repressive effect on *MYL2*, and plays a role in the occurrence and development of certain cancers [16]. However, the exploration of lncRNAs as ceRNAs in bovine mastitis is still in its infancy.

MicroRNAs (miRNAs) are small (18–25 nt) stable non-coding RNAs that regulate gene expression post-transcriptionally by binding target mRNAs and affecting their translation and stability [17,18]. As core components of ceRNA networks, miRNAs are pivotal regulators in diverse immune and inflammatory contexts [19]. In cattle, miR-145 has been described as an inflammation-related miRNA [20,21,22], and its altered expression has been reported in bovine mammary tissues during *S. aureus* infection [23,24]. Functional studies indicate that miR-145 can influence BMEC proliferation by targeting genes like *FSCN1* and *IRS1* [23,25]. Together, these findings suggest that miR-145 may contribute to mastitis-related epithelial responses.

Previous bioinformatic analyses have proposed *ANO6* as a potential miR-145 target [26]. *ANO6*, also known as transmembrane protein 16F (TMEM16F), is a TMEM16 family member with ion channel-related functions and phospholipid scramblase activity [27,28,29]. It has been reported to participate in immune responses, apoptosis and cell fusion processes, and it plays a key role in cell membrane repair [28]. In the context of regulated cell death, *ANO6* has been implicated in pathways related to pyroptosis and necrotic cell death, suggesting a role in shaping cell fate and tissue homeostasis [30]. In immune settings, *ANO6* has also been associated with T-cell activation, macrophage phagocytic activity, and inflammatory mediator release [31]. Collectively, these studies suggest that *ANO6* may function in pathological conditions accompanied by cellular stress and injury, yet its relevance in bovine mastitis has not been directly established.

In this study, we established a *S. aureus*-induced bovine mastitis model. Through transcriptomic profiling and functional screening, we identified a novel long non-coding RNA, BMNCR, which exhibits specific upregulation in mastitis-affected mammary tissues. Mechanistic investigations suggest a model in which BMNCR may modulate inflammatory responses and epithelial cell responses in BMECs potentially via a miR-145/*ANO6* axis. These findings deepen our understanding of epigenetic regulation in bovine mastitis and highlight BMNCR as a candidate protective regulator and potential biomarker for *S. aureus*-induced mastitis.

## 2. Materials and Methods

### 2.1. Experimental Animals and Ethics Statement

Three healthy primiparous Holstein cows (half-siblings) in mid-lactation were selected from the farm of Yangzhou University. The cows had similar body weights, parity, age, and milk production levels, with SCC below 100,000 cells/mL and no history of mastitis. After a one-week acclimation period under standardized conditions, the experiment was initiated.

All procedures were approved by the Institutional Animal Care and Use Committee of Yangzhou University (SYXK (SU)2022-0044) and conducted in compliance with China’s national guidelines for experimental animal welfare (Ministry of Science and Technology).

### 2.2. Mastitis Model Construction

*S. aureus* (ATCC 25923) was cultured on solid Luria–Bertani (LB) medium at 37 °C for 24 h. A single colony was subsequently inoculated into 2 mL of liquid LB medium and incubated overnight at 37 °C. Bacterial suspensions were adjusted to a concentration of 1 × 10^7^ CFU/mL using sterile phosphate-buffered saline (PBS) (Biosharp, Hefei, China). The right front mammary quarters of each cow were infused with 5 mL of the bacterial suspension, while the left front quarters received 5 mL of sterile PBS and served as negative controls. Milk yield, SCC, and body temperature were recorded. Successful infection was confirmed 24 h post-inoculation based on the presentation of clinical signs. Mammary tissue samples were surgically collected for histopathological examination using hematoxylin and eosin (H&E) staining to verify the induction of mastitis. All samples were flash-frozen in liquid nitrogen and stored at −80 °C until further analysis.

### 2.3. RNA Extraction and Sequencing

Total RNA was extracted from mammary gland tissues using the mirVana^TM^ RNA Isolation Kit (Applied Biosystems, Foster City, CA, USA) and further purified using the RNeasy Kit (QIAGEN, Hilden, Germany). RNA integrity was evaluated using an Agilent 2100 Bioanalyzer (Agilent Technologies, Santa Clara, CA, USA), and only RNA samples with an RNA Integrity Number >7 were selected for sequencing. High-quality RNA (5 µg) was sent to Oebiotech (Shanghai, China) for small RNA library construction and high-throughput sequencing on the Illumina platform.

### 2.4. CeRNA Network Analysis

Initial prediction of miRNA-target interactions (miRNA-mRNA or miRNA-lncRNA) was conducted using miRanda software (v3.3a) to identify potential microRNA recognition sites in genomic sequences. Subsequently, lncRNA-mRNA regulatory pairs were predicted based on shared miRNA binding partners. To enhance prediction reliability, correlation analysis of expression values was performed for each regulatory pair. MiRNA-target relationships were filtered using dual criteria: Pearson correlation coefficient (PCC) absolute value ≥0.7, and associated *p*-value ≤ 0.05 derived from expression profile correlations. Final network visualization was implemented in the R software environment (v4.2.1) with igraph and ggplot2 packages.

### 2.5. Isolation and Culture of Cells

Primary BMECs were isolated from healthy lactating bovine mammary tissue using enzymatic digestion (the identity of the primary cells was confirmed as shown in Figure S2, CK18 immunofluorescent staining and morphological analysis). Bovine mammary alveolar cell-T (Mac-T) cells were provided by the College of Veterinary Medicine, Yangzhou University. Cells were cultured at 37 °C with 5% CO_2_ in complete growth medium containing DMEM/F12 (Gibco, Waltham, MA, USA) supplemented with 10% fetal bovine serum (FBS, Gibco, Waltham, MA, USA), 100 U/mL penicillin, and 100 ug/mL streptomycin (Invitrogen, Carlsbad, CA, USA).

### 2.6. Heat-Inactivated S. aureus Stimulation of BMECs

Primary BMECs were seeded in 6-well plates and cultured to approximately 70–80% confluence. The *S. aureus* suspension prepared in Section 2.2 was heat-inactivated at 80 °C for 30 min. Before stimulation, the culture medium was replaced with antibiotic-free DMEM/F12 complete medium. The heat-inactivated bacterial suspension was then diluted in this medium and added to BMECs at a multiplicity of infection (MOI) of 100:1 according to the predetermined bacterial concentration and the number of BMECs. Control cells were cultured in the same medium without bacterial stimulation. After 6 h of incubation [32], cells were collected for RNA extraction and qRT-PCR analysis. Each treatment was performed in triplicate.

### 2.7. Transfection

Sub-confluent BMECs were transfected with small RNA mimics or inhibitors using Lipofectamine 2000 (Invitrogen, USA). Experimental procedures were initiated 48 h post-transfection, with each treatment condition performed in triplicate.

Gene-specific siRNAs targeting BMNCR (siBMNCR-1535/161/2554) and *ANO6* (siANO6-1507/2812/1041), along with scrambled siRNA control (si-NC), were synthesized by GenePharma (Shanghai, China). The miR-145 mimic, inhibitor, and corresponding negative controls (mimic-NC, inhibitor-NC) were obtained from the same vendor. All primers for qPCR analysis, including targets, inflammatory cytokines, reference genes, candidate miRNAs and potential target genes, were designed via Primer Premier 5.0 and synthesized by GenePharma, with sequences provided in Tables S1–S3.

### 2.8. RNA Isolation and qRT-PCR Assay

Total RNA was isolated using TRIzol reagent (Vazyme, Nanjing, China) and reverse-transcribed with HiScript qRT SuperMix (Vazyme, Nanjing, China) following manufacturer protocols. Quantitative PCR was performed using AceQ SYBR Master Mix (Vazyme, Nanjing, China). GAPDH, β-actin and U6 were used as internal controls. All reactions were repeated in three independent experiments, and relative expression levels were calculated using the 2^−ΔΔCt^ method.

### 2.9. Subcellular Localization Analysis

Nuclear and cytoplasmic fractions of BMECs were prepared using the PARIS™ Kit (Thermo Fisher Scientific, Waltham, MA, USA). RNA from separated fractions underwent qRT-PCR using primers specific to BMNCR, with GAPDH (cytoplasmic control) and U6 (nuclear control) for normalization.

### 2.10. Western Blot

Cellular proteins were extracted using RIPA lysis buffer (Beyotime Biotechnology, Shanghai, China) and quantified via BCA assay. Proteins were then immunoblotted using primary antibodies against ANO6 (Abcam, Cambridge, UK) and β-actin (Abcam). Anti-rabbit IgG-HRP (Sungene Biotech, Tianjin, China) was used as a secondary antibody. The bands on the blot were visualized using ECL chemiluminescent substrate (Vazyme, Nanjing, China), and their intensities were quantified with ImageJ software (version 1.44p).

### 2.11. Cell Apoptosis and Proliferation Assays

Cell apoptosis was detected using an Annexin V-FITC/PI double-staining kit (Vazyme, Nanjing, China). Fluorescence signals were detected using a CytoFLEX S flow cytometer (Beckman Coulter, Brea, CA, USA) and analyzed with CytExpert software (version 2.3.1). All procedures were performed under light-protected conditions, with a staining duration of 10 min.

Cell proliferation was evaluated using the BeyoClick^TM^ EdU Cell Proliferation Detection Kit (Beyotime, Shanghai, China) combined with the CCK-8 Assay Kit (C0037, Beyotime, Shanghai, China). For EdU labeling, cells were imaged using a Leica DMi8 inverted fluorescence microscope (Leica Microsystems, Wetzlar, Germany). The CCK-8 assay involved incubating cells with CCK-8 reagent (10% of the total culture volume) at 37 °C for 1 h, followed by absorbance measurement at 450 nm.

### 2.12. Dual-Luciferase Reporter Assay

Putative binding interactions were validated with dual-luciferase reporter systems. Wild-type (WT) and mutant (MUT) sequences of BMNCR and *ANO6* containing miR-145 binding sites were cloned into psiCHECKTM-2 (Promega, Madison, WI, USA) vectors. Endofree plasmid kit was used to extract plasmid. Constructs were co-transfected with miR-145 mimic or negative control into 293T cells using Lipofectamine 2000. Luciferase activity was determined by Dual Luciferase Assay Kit (Vazyme Biotech, Nanjing, China) in line with the manufacturer’s instructions.

### 2.13. Statistical Analysis

Data were analyzed using Excel, and visualized with GraphPad 8.0, ImageJ, and R 4.2. Group differences were assessed using Student’s *t*-test for two groups or one-way analysis of variance (ANOVA) for more than two groups. Data are expressed as mean ± SEM unless otherwise noted. Statistical significance was set at *p* < 0.05 (* *p* < 0.05, ** *p* < 0.01).

## 3. Results

### 3.1. Transcriptomic Profiling Reveals S. aureus-Induced ceRNA Networks in Bovine Mastitis

An experimental mastitis model was successfully established by infecting bovine mammary glands with *S. aureus*. Within 24 h post-inoculation, all three infected cows exhibited clinical signs such as anorexia, tachypnea, pyrexia, and marked redness and swelling of the mammary glands (Figure S1A). Concurrently, SCC in milk increased significantly (SCC > 2,000,000 cells/mL, Figure S1B). H&E staining further confirmed the development of mastitis (Figure S1C).

**Figure 1:**
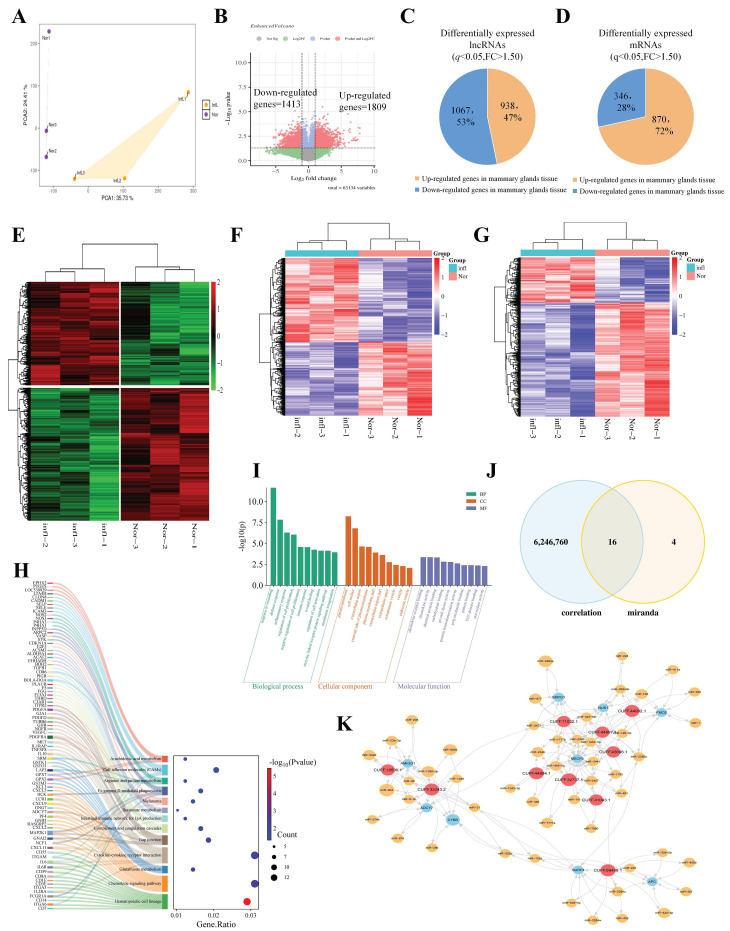
Integrated network inference identifies key lncRNAs in *S. aureus*-induced bovine mastitis. (**A**) PCA score plots of all samples. (**B**) Volcano plot showing differentially expressed genes (DEGs) with fold change (FC) versus adjusted *p*-value. (**C**,**D**) Pie charts depicting proportions of differentially expressed lncRNAs (**C**) and mRNAs (**D**) in *S. aureus*-infected mammary tissues (RNA-seq, *p* < 0.05, *FC* > 1.5). (**E**) Heatmap of DEGs. (**F**,**G**) Heatmaps showing expression profiles of differentially expressed lncRNAs (**F**) and mRNAs (**G**). (**H**) KEGG pathway enrichment of DEGs. (**I**) GO analysis of DEGs, including biological processes (BP), cellular components (CC), and molecular functions (MF). (**J**) Venn diagram integrating ceRNA network predictions and expression correlations, while the blue section indicates correlations derived from experimental expression data. (**K**) ceRNA regulatory network diagram, red represents lncRNA, blue represents genes, and yellow represents miRNA. The data were shown as the mean ± SEM (*n* = 3).

To identify dysregulated lncRNAs in *S. aureus*-infected mammary tissues, high-throughput RNA sequencing was performed on three paired bovine mammary tissue samples. Principal component analysis (PCA) revealed pronounced segregation between infected and control groups (Figure 1A). Comparative transcriptomics identified 3222 differentially expressed RefSeq genes (Figure 1E), including 1809 upregulated and 1413 downregulated transcripts (Figure 1B). Notably, 870 mRNAs and 938 lncRNAs were upregulated, while 346 mRNAs and 1067 lncRNAs were downregulated in *S. aureus*-infected tissues (Figure 1C,D). Hierarchical clustering revealed distinct expression patterns for both mRNAs (Figure 1F) and lncRNAs (Figure 1G). Gene Ontology enrichment analysis of differentially expressed genes (DEGs) highlighted their significant association with mastitis-related biological processes, including inflammatory response, cell proliferation, immune response, and regulation of cell activation (Figure 1I). Kyoto Encyclopedia of Genes and Genomes (KEGG) pathway analysis further identified 14 enriched pathways, spanning immune regulation, metabolic adaptation, and disease-related mechanisms (Figure 1H). To investigate the ceRNA regulatory network underlying *S. aureus* mastitis, we identified 16 lncRNAs with potential ceRNA activity through systematic ceRNA network analysis (Figure 1J). The top 10 most robust lncRNA-mRNA pairs were subsequently selected to construct the ceRNA regulatory network diagram (Figure 1K).

### 3.2. BMNCR Expression Is Upregulated in S. aureus-Induced Mastitis Tissue and Involved in the Proliferation, Apoptosis, and Inflammatory Response of BMECs

**Figure 2:**
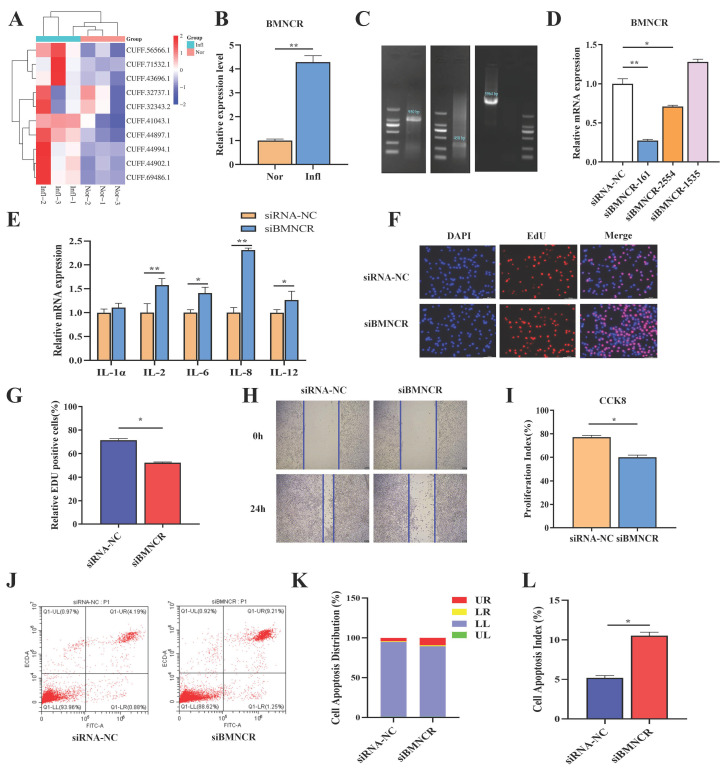
BMNCR regulates proliferation, apoptosis, and inflammation in BMECs. (**A**) qRT-PCR analysis of 10 differentially expressed lncRNAs in *S. aureus*-stimulated BMECs versus healthy BMECs. (**B**) BMNCR expression in *S. aureus*-stimulated BMECs measured by qRT-PCR. (**C**) Full-length BMNCR sequence obtained via RACE. (**D**) BMNCR knockdown efficiency of three siRNAs by qRT-PCR. (**E**) Inflammatory cytokine levels in siBMNCR-transfected BMECs. (**F**) Proliferation assessed by EdU assay. (**G**) Quantification of EdU-positive cells using ImageJ. (**H**) Cell migration measured by scratch assay (Scale bar = 100 µm). (**I**) Cell viability via CCK-8 assay. (**J**) Apoptosis detection by Annexin V-FITC/PI dual staining and flow cytometry. (**K**) Apoptotic cell distribution profiles in siRNA-transfected BMECs. (**L**) Apoptosis index post-siBMNCR transfection in BMECs (Apoptotic index calculated as the percentage of early and late apoptotic cells). The data were shown as the mean ± SEM (*n* = 3) * *p* < 0.05, ** *p* < 0.01.

To validate whether the identified candidate lncRNAs possess significant function, we analyzed the expression of 10 differentially expressed lncRNAs in *S. aureus*-stimulated BMECs and normal BMECs using qRT-PCR (Figure 2A). The data revealed that the expression of CUFF.43696.1 was significantly upregulated in *S. aureus*-stimulated BMECs (*p* < 0.01), and we renamed it BMNCR (Figure 2B). In the current study, we focused on investigating the potential functional role of BMNCR in inflammatory responses. Using the SMARTer RACE cDNA Amplification Kit, we amplified the 5′ and 3′ ends of BMNCR and obtained its full-length sequence of 5964 nt (Figure 2C, Supplementary Data). To further examine the function of BMNCR in BMECs, we transfected BMECs with three siRNAs targeting BMNCR. qRT-PCR analysis showed that siBMNCR-161 (siBMNCR) significantly downregulated BMNCR expression (*p* < 0.01) (Figure 2D). Following BMNCR knockdown, certain inflammation-related cytokines were modulated (Figure 2E), with IL-2, IL-6, IL-8, and IL-12 expression increasing (*p* < 0.05, *p* < 0.01), while IL-1α expression remained unchanged. EdU analysis demonstrated that BMNCR depletion reduced the number of EdU-positive cells (Figure 2F,G), indicating significantly inhibited proliferation in BMECs (*p* < 0.05). The scratch assay revealed that BMNCR knockdown suppressed the migration of BMECs (Figure 2H). CCK-8 assay results showed that siBMNCR markedly suppressed the proliferative activity of BMECs (Figure 2I). Similarly, flow cytometry results indicated that siBMNCR increased the apoptosis rate of BMECs (Figure 2J,K) and significantly raised the apoptosis index (*p* < 0.01) (Figure 2L).

### 3.3. BMNCR Is Located in the Cytoplasm and Regulates miR-145 Expression

**Figure 3:**
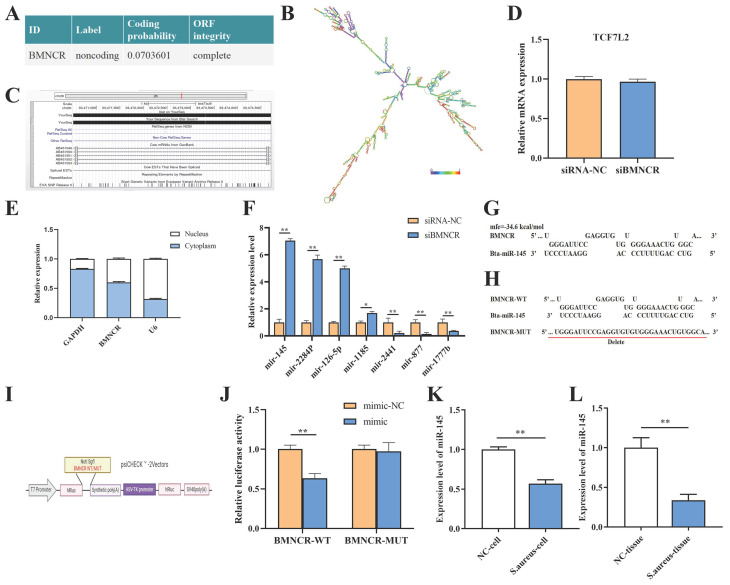
Subcellular localization and miR-145 interaction of BMNCR. (**A**) CPC2 validation of BMNCR as a lncRNA. (**B**) Predicted secondary structure of BMNCR. (**C**) BMNCR is located in the third intron of *TCF7L2* on bovine chromosome 26, as shown by UCSC Genome Browser alignment. (**D**) *TCF7L2* expression unaffected by BMNCR knockdown. (**E**) Nuclear/cytoplasmic distribution of BMNCR (qRT-PCR). (**F**) Candidate miRNAs targeting BMNCR validated by qRT-PCR. (**G**) Predicted miR-145 binding sites in BMNCR. (**H**) Schematic of BMNCR WT/MUT constructs. (**I**) WT/MUT BMNCR fragments cloned into psiCHECK2. (**J**) Dual-luciferase reporter assay to evaluate the binding capacity between BMNCR and miR-145. (**K**) miR-145 expression is significantly downregulated in *S. aureus*-infected bovine mammary tissues. (**L**) miR-145 expression is suppressed in *S. aureus*-stimulated BMECs. The data were shown as the mean ± SEM (*n* = 3) * *p* < 0.05, ** *p* < 0.01.

To elucidate the regulatory mechanisms of BMNCR in bovine mastitis, we first employed the Coding Potential Calculator (CPC) to assess its coding potential, which confirmed BMNCR as a novel unannotated lncRNA (Figure 3A). Subsequent analysis with NCBI ORFfinder identified 39 open reading frames (ORFs) in BMNCR, but none exhibited coding potential (Figure S3). Secondary structure prediction via the RNAfold network server further revealed that BMNCR folds into a stable conformation characterized by multiple hairpin loops (Figure 3B). Alignment via the UCSC Genome Browser showed BMNCR is in the third intron of *TCF7L2* on bovine chromosome 26 (Figure 3C), and BMNCR knockdown does not significantly affect *TCF7L2* expression (Figure 3D). The regulatory patterns of lncRNAs are closely related to their subcellular localization. Therefore, we performed nuclear-cytoplasmic fractionation on BMECs and used qRT-PCR analysis to assess the subcellular localization of BMNCR in BMECs. The results showed that BMNCR could be observed in both the nucleus and cytoplasm of BMECs (Figure 3E), which suggested that BMNCR may exert part of its biological function through a ceRNA-related mechanism.

Using ceRNA network analysis, RNAhybrid, and miRanda, we identified miRNAs potentially targeting BMNCR. Given the study’s focus on immune function and inflammation, miR-145, miR-2284p, miR-126-5p, miR-1185, miR-2441, miR-877, and miR-1777b were selected for qRT-PCR validation based on NCBI functional annotation (Figure 3F). Among these, miR-145 exhibited the most significant upregulation following BMNCR knockdown. RNAhybrid prediction revealed potential binding site between BMNCR and miR-145 (Figure 3G). To validate this interaction, dual-luciferase reporter assays were performed using psi-Check2 vectors containing BMNCR-WT or BMNCR-MUT constructs (Figure 3I). The BMNCR-MUT construct was generated by targeted deletion of the miR-145 seed-complementary region in the BMNCR sequence (Figure 3H). Co-transfection with the miR-145 mimic significantly reduced luciferase activity in BMNCR-WT by 37% (*p* < 0.01), whereas no change was observed in BMNCR-MUT (Figure 3J). qRT-PCR analysis of *S. aureus*-infected bovine mammary tissues and *S. aureus*-stimulated BMECs further confirmed these findings (Figure 3K,L). Both sample types showed significant downregulation of miR-145, revealing a negative regulatory relationship between miR-145 expression and *S. aureus* infection.

### 3.4. Functional Analysis of miR-145 in BMECs

**Figure 4:**
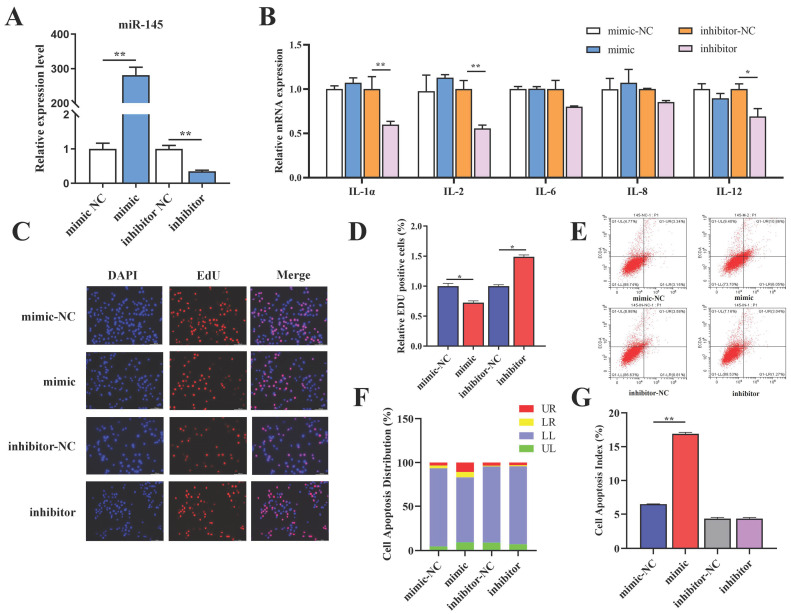
Functional characterization of miR-145 in BMECs. (**A**) Validation of miR-145 overexpression or knockdown efficiency by qRT-PCR. (**B**) Inflammatory cytokine profiles post miR-145 modulation. (**C**) Proliferation assessed by EdU assay. (**D**) Quantification of EdU-positive cells using ImageJ. (**E**) Apoptosis detection by Annexin V-FITC/PI dual staining and flow cytometry. (**F**) Apoptotic cell distribution profiles. (**G**) Apoptotic index. The data were shown as the mean ± SEM (*n* = 3) * *p* < 0.05, ** *p* < 0.01.

To investigate the regulatory role of miR-145 in BMECs, transient transfection of miR-145 mimic or inhibitor was performed using Lipofectamine 2000. qRT-PCR validation confirmed significant upregulation of miR-145 in mimic-treated cells and significant downregulation in inhibitor-treated groups (*p* < 0.01) (Figure 4A). Functional assays revealed miR-145’s role in inflammation regulation: miR-145 mimic transfection did not alter pro-inflammatory cytokine levels, while miR-145 inhibitor transfection suppressed IL-1α, IL-2, and IL-12 expression (*p* < 0.05, *p* < 0.01) (Figure 4B). Proliferation and apoptosis analyses demonstrated miR-145’s growth-inhibitory effects. EdU incorporation assays showed reduced proliferation in BMECs transfected with the miR-145 mimic, while increased proliferation in cells treated with the miR-145 inhibitor (Figure 4C,D). Apoptosis assays revealed a significant increase in cell death in miR-145 mimic-transfected BMECs (Figure 4E–G), whereas miR-145 inhibitor-treated cells exhibited no apoptotic effects. Collectively, these findings indicate that miR-145 inhibits BMEC proliferation and promotes apoptosis.

### 3.5. Bioinformatic Prediction and Mechanistic Validation Identify ANO6 as a Functional Target of miR-145 Modulated via BMNCR ceRNA Activity

**Figure 5:**
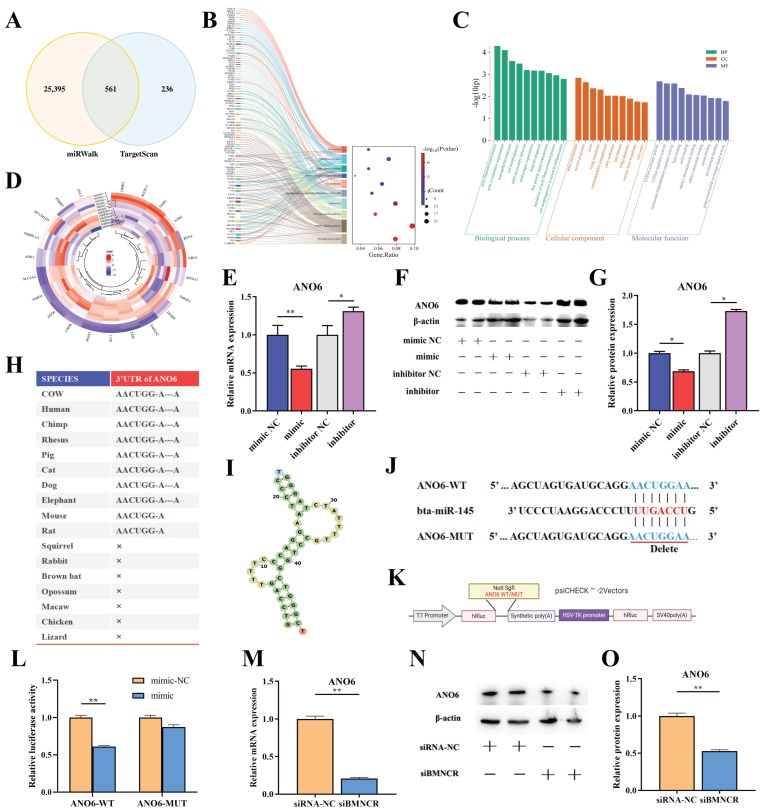
*ANO6* is a functional target of miR-145 in the BMNCR ceRNA axis. (**A**) Overlap of miR-145 targets predicted by miRWalk and TargetScan. (**B**) KEGG pathway enrichment analysis of miR-145 targets. (**C**) GO analysis of miR-145 targets. (**D**) qRT-PCR validation of 23 prioritized miR-145 targets. (**E**) *ANO6* mRNA levels inversely regulated by miR-145 mimic and inhibitor. (**F**) ANO6 protein expression detected by Western blot. (**G**) Densitometric quantification of ANO6 protein. (**H**) Evolutionary conservation of miR-145 binding sites in the *ANO6* 3′-UTR across 10 species (TargetScan). (**I**) Predicted secondary structure of the miR-145/*ANO6* binding region. (**J**) Schematic of *ANO6* 3′-UTR WT/MUT constructs. (**K**) Cloning strategy for WT/MUT *ANO6* into psiCHECK2. (**L**) Dual-luciferase reporter assay to evaluate the binding capacity between *ANO6* and miR-145. (**M**) *ANO6* mRNA suppression by BMNCR knockdown. (**N**) ANO6 protein reduction in BMNCR-silenced BMECs. (**O**) Densitometric analyses of ANO6 protein levels post-BMNCR knockdown. The data were shown as the mean ± SEM (*n* = 3) * *p* < 0.05, ** *p* < 0.01.

Using miRWalk3.0 and TargetScan8.0, we predicted 561 potential downstream targets of miR-145 (Figure 5A). KEGG pathway enrichment analysis revealed these genes were significantly enriched in 57 KEGG pathways (Figure 5B), including cancer-related signaling pathways such as Proteoglycans in cancer, MAPK signaling pathway, TGF-beta signaling pathway, and Hippo signaling pathway; cellular processes like endocytosis and focal adhesion; and immune responses exemplified by bacterial invasion of epithelial cells. Gene Ontology analysis identified 192 biological processes (e.g., cell motility, division), 28 cellular components, and 17 molecular functions (Figure 5C). These enriched GO and KEGG pathways may play crucial roles in the pathogenesis of bovine *S. aureus* mastitis.

Twenty-three genes were prioritized based on functional annotation from the NCBI database to construct a miR-145–mRNA interaction network (Figure 5D). Among them, *ANO6* was selected for further validation because it showed consistent inverse regulation by miR-145 and has a known role in plasma membrane repair, which is relevant to epithelial integrity during mastitis. To determine whether this functionally relevant gene is a target of miR-145, we examined their regulatory relationship. We found that *ANO6* expression was inversely regulated by miR-145, with mimic transfection suppressing and inhibitor treatment elevating its mRNA levels (Figure 5E). Western blot (Figure 5F) and densitometric analyses (Figure 5G) confirmed concordant protein-level changes, prompting further investigation of ANO6. Bioinformatic prediction identified evolutionarily conserved binding sites for miR-145 within the *ANO6* 3′-UTR (Figure 5H,I). The binding capacity between *ANO6* and miR-145 was assessed by deleting *ANO6*’s seed region using the psi-Check2 vector (Figure 5J,K). In the dual-luciferase reporter assay (Figure 5L), transfection of miR-145 mimic significantly reduced luciferase activity by 40% in BMECs transfected with *ANO6*-WT (*p* < 0.01), supporting that *ANO6* is a direct target of miR-145.

To investigate whether BMNCR regulates *ANO6* through ceRNA activity, we assessed *ANO6* expression dynamics under BMNCR perturbation. qRT-PCR revealed that BMNCR knockdown suppressed *ANO6* mRNA levels (*p* < 0.01) (Figure 5M), while concurrent Western blot and densitometric analyses demonstrated a corresponding attenuation of ANO6 protein expression (*p* < 0.01) (Figure 5N,O). Notably, the suppression of *ANO6* induced by BMNCR knockdown was effectively rescued by co-transfection with a miR-145 inhibitor (Figure S4). We performed parallel experiments in MAC-T cells, which consistently showed that BMNCR knockdown led to decreased expression of *ANO6* at mRNA levels (Figure S5). These results corroborate the regulatory relationship between BMNCR and *ANO6* across different cellular models and support the conclusion that BMNCR modulates *ANO6* expression by sponging miR-145.

### 3.6. Functional Validation of ANO6 in BMNCR-Mediated Regulation of BMECs

**Figure 6:**
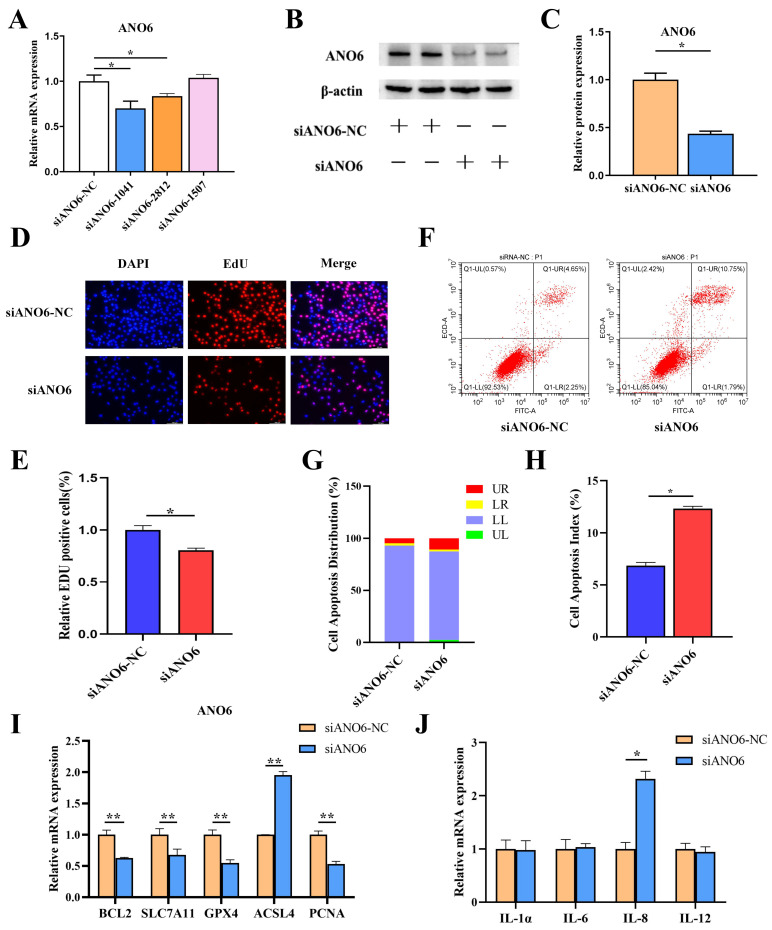
*ANO6* mediates BMNCR regulatory functions in BMECs. (**A**) *ANO6* mRNA levels in BMECs transfected with three independent siRNAs, quantified by qRT-PCR. (**B**) ANO6 protein expression detected by Western blot post-siANO6-1041 transfection. (**C**) Densitometric analysis of ANO6 protein levels after siANO6 transfection. (**D**) Proliferation capacity of BMECs assessed by EdU incorporation assay. (**E**) Quantification of EdU-positive cells by ImageJ. (**F**) Apoptosis analysis by Annexin V-FITC/PI dual staining and flow cytometry. (**G**) Apoptotic cell distribution profiles. (**H**) Apoptotic index. (**I**) Effects of *ANO6* knockdown on the expression of genes associated with apoptosis, oxidative stress, and lipid metabolism in BMECs. (**J**) IL-8 upregulation in siANO6-treated BMECs. The data were shown as the mean ± SEM (*n* = 3) * *p* < 0.05, ** *p* < 0.01.

To evaluate *ANO6*’s role in BMNCR-mediated regulation, three siRNAs targeting *ANO6* were transfected into BMECs. qRT-PCR showed that *ANO6* mRNA expression was significantly downregulated (*p* < 0.05) after transfection with siANO6-1041 (siANO6) (Figure 6A). Western blot and densitometric analyses confirmed reduced ANO6 protein levels after siANO6 transfection (*p* < 0.01) (Figure 6B,C). Functional assays demonstrated *ANO6*’s dual role in cellular homeostasis: EdU incorporation assays revealed a substantial decrease in proliferating BMECs (Figure 6D,E), while Annexin V/PI staining showed a significant increase in apoptosis (Figure 6F–H). To further explore downstream molecular changes associated with *ANO6* knockdown, we examined several genes related to proliferation, apoptosis, oxidative stress, and lipid metabolism. *ANO6* knockdown significantly increased ACSL4 expression (*p* < 0.01), while significantly reducing expression of BCL2, SLC7A11, GPX4, and PCNA (*p* < 0.01) (Figure 6I). In addition, *ANO6* knockdown significantly increased IL-8 expression (*p* < 0.05) (Figure 6J). These findings indicate that *ANO6* is involved in BMNCR-associated regulation of proliferation, apoptosis, inflammatory factor expression, and related downstream molecular changes in BMECs.

## 4. Discussion

Recent advances in epigenetic studies have positioned lncRNAs as pivotal regulators in bovine mastitis pathogenesis, with accumulating evidence demonstrating their critical roles in modulating apoptosis, proliferation, and inflammatory responses within BMECs [33,34]. For instance, the highly expressed lncRNA XIST suppresses NF-κB pathway activation and NLRP3 inflammasome assembly through a negative feedback mechanism, consequently attenuating pro-inflammatory cytokine secretion in MAC-T cells [35]. However, the functional significance and regulatory networks of aberrantly expressed lncRNAs in bovine mastitis have yet to be fully deciphered. Since the proposal of the ceRNA hypothesis, therapeutic targets and biomarkers for bovine mastitis have been continuously identified, offering critical insights and research directions to advance early diagnosis and clinical management of the disease. A representative example includes lncRNA CA12-AS1, which exacerbates lipopolysaccharide (LPS)-induced inflammatory responses in BMECs by sponging miR-133a, thereby modulating the expression of proliferation-associated genes such as *PCNA* and *CDK4* [15]. To further explore these mechanisms, we established *S. aureus*-induced bovine mastitis models and performed transcriptomic and functional analyses using bovine mammary tissues and BMECs [23]. Transcriptomic profiling identified 2005 differentially expressed lncRNAs potentially associated with bovine mastitis progression. Subsequent functional enrichment analysis revealed their predominant involvement in inflammatory response, cellular proliferation, and immune regulation. Based on these findings, we systematically constructed a ceRNA regulatory network specific to *S. aureus*-induced bovine mastitis, through which the BMNCR was preliminarily identified as a candidate regulator exhibiting significant upregulation in infected mammary tissues. Subsequent validation experiments demonstrated that BMNCR expression was remarkably upregulated in *S. aureus*-stimulated BMECs. Functional characterization demonstrated that BMNCR knockdown significantly attenuated cellular proliferation while augmenting apoptotic rates. Importantly, BMNCR knockdown exerted no significant effect on *TCF7L2* expression, consistent with its intronic localization within the *TCF7L2* gene. Subcellular localization assays confirmed BMNCR’s cytoplasmic predominance, suggesting its functional relevance to ceRNA-mediated post-transcriptional regulation [36]. Moreover, in vitro analyses demonstrated that BMNCR knockdown altered the expression of interleukin family cytokines (e.g., IL-2, IL-6, IL-8, and IL-12), which are important mediators of inflammatory responses involved in host defense against pathogenic insults [37]. Taken together, the observed expression pattern and knockdown phenotypes suggest that BMNCR exerts a protective effect on epithelial homeostasis, whereas its upregulation in *S. aureus*-associated tissues and cells may reflect a compensatory response to inflammatory injury. This interpretation is supported by previous reports showing that some inflammation-associated lncRNAs are upregulated during inflammatory activation and contribute to feedback control of excessive inflammatory injury, including XIST in bovine mammary epithelial cells and Mirt2 [38] and LUCAT1 [39] in other inflammatory models. Collectively, our findings support a protective/compensatory biological role of BMNCR in *S. aureus*-induced bovine mastitis.

The rapid advancement of bioinformatics has established a methodological foundation for investigating how lncRNAs regulate target gene expression through the ceRNA mechanism. In this study, we identified miR-145 binding sites within the BMNCR sequence using bioinformatics analysis and subsequently validated their interaction through dual-luciferase reporter assays. Our findings revealed that BMNCR negatively regulates miR-145 expression. Notably, miR-145 expression was significantly downregulated in *S. aureus*-stimulated BMECs, an observation consistent with previous reports by Chen et al. [40]. Prior studies have established that miR-145, an immune-related miRNA [41], plays critical roles in NF-κB signaling-mediated immune responses by reducing oxidative stress, suppressing apoptosis, and downregulating NF-κB/p65 signaling [42,43]. Furthermore, miR-145 has been implicated in mammary gland development, milk fat synthesis regulation, and cancer pathogenesis [44,45]. Emerging evidence suggests its involvement in bovine mastitis, with decreased miR-145 levels observed in mastitic tissues and demonstrated regulatory effects on key signaling pathways including AKT/GSK and NF-κB [46]. Specifically in the context of *S. aureus*-induced mastitis, miR-145 has been shown to influence BMEC functionality by targeting *FSCN1* [40]. Through KEGG and GO enrichment analyses of miR-145 target genes, we elucidated their regulatory roles in cellular processes, cancer-related pathways, and immune responses. Functional experiments demonstrated that miR-145 overexpression significantly reduced BMECs proliferation and enhanced apoptosis, with no significant changes in pro-inflammatory cytokines in BMECs. Intriguingly, although miR-145 knockdown did not alter cellular apoptosis, it markedly suppressed the expression of pro-inflammatory cytokines IL-1α, IL-2, and IL-12 in BMECs. Collectively, our study demonstrates that BMNCR is involved in *S. aureus*-induced bovine mastitis through modulation of miR-145 activity.

To explore potential downstream effectors of miR-145 within the BMNCR regulatory axis, we examined 23 candidate genes based on target prediction and functional annotation. Among these candidates, *ANO6* was negatively regulated by miR-145 in BMECs, with miR-145 mimic suppressing and miR-145 inhibitor increasing *ANO6* expression at both the mRNA and protein levels. *ANO6* is a phospholipid scramblase involved in volume regulation, phospholipid scrambling and plasma membrane repair [47], functions that are relevant to how mammary epithelial cells respond to inflammatory stress. We further supported direct targeting of *ANO6* by miR-145 using dual-luciferase reporter assays, showing that miR-145 reduced luciferase activity from the *ANO6* 3′-UTR WT construct and that disruption of the predicted binding site attenuated this effect. Consistent with the miR-145/ANO6 targeting results, BMNCR knockdown reduced *ANO6* expression at both mRNA and protein levels in BMECs, and this reduction was reversed by co-transfection with a miR-145 inhibitor. This rescue result is consistent with a BMNCR/miR-145/*ANO6* regulatory relationship, where BMNCR affects *ANO6* by modulating miR-145 (Figure S4). In addition, *ANO6* silencing partly recapitulated the effects observed after BMNCR knockdown, including reduced cell proliferation, increased apoptosis, and elevated IL-8 expression. However, the downstream inflammatory phenotypes were not fully consistent across BMNCR, miR-145, and *ANO6*. BMNCR knockdown altered multiple interleukin-family cytokines, whereas miR-145 and *ANO6* modulation showed more limited effects on these inflammatory readouts. This suggests that, although miR-145 and *ANO6* are validated components of the BMNCR-associated regulatory network in BMECs, the broader inflammatory changes associated with BMNCR knockdown are not fully explained by this single route. This interpretation is reasonable because ceRNA regulation does not operate through a simple linear pathway and instead occurs within a broader competitive post-transcriptional network, where the downstream output may vary with the relative abundance of endogenous miRNAs and their target transcripts [48,49]. Therefore, in addition to the BMNCR/miR-145/*ANO6* pathway identified here, BMNCR may also participate in inflammatory regulation through other downstream routes. Taken together, these results place miR-145 and *ANO6* as key downstream components of BMNCR and support the involvement of this regulatory axis in BMNCR-associated control of proliferation, apoptosis, and inflammatory responses in BMECs.

What *ANO6* does after it is regulated in the context of bovine mastitis is still not fully defined. Recent studies in breast cancer suggest that *ANO6* activation can participate in regulated cell death programs and has been discussed in relation to ferroptosis-related signaling [50,51]. In mammary inflammatory models, ferroptosis-associated changes have also been reported during epithelial injury [52], and blocking ferroptosis-related processes was shown to alleviate epithelial and tissue damage in LPS- or S. aureus-induced mastitis models [53,54]. Based on these observations, we speculated that *ANO6* may be linked to ferroptosis-related transcriptional changes and therefore performed an exploratory analysis after *ANO6* knockdown in BMECs. *ANO6* knockdown decreased the expression of PCNA, BCL2, GPX4, and SLC7A11, while increasing ACSL4 expression. These changes indicate that *ANO6* knockdown alters the transcriptional expression of genes associated with cell survival, oxidative stress, and lipid metabolism. However, the current data are limited to selected transcriptional markers, and the downstream mechanisms through which *ANO6* contributes to mammary epithelial responses during inflammatory challenge remain to be further clarified.

**Figure 7:**
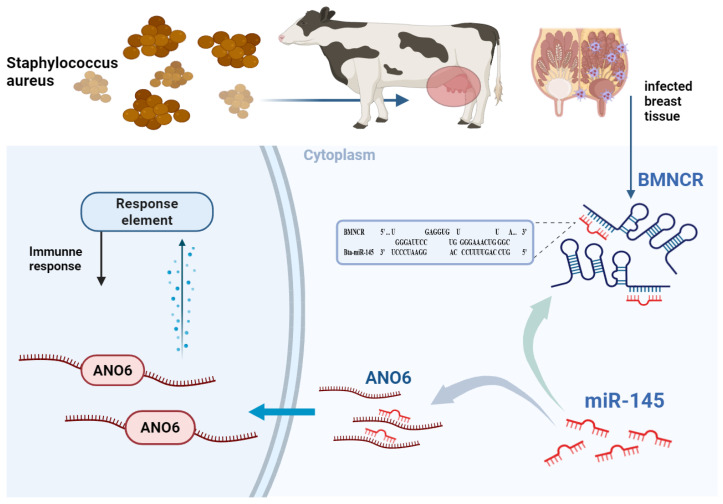
Proposed model of the BMNCR/miR-145/*ANO6* ceRNA axis in BMECs.

This study reveals a novel regulatory relationship of the BMNCR/miR-145/*ANO6* axis in *S. aureus*-induced bovine mastitis (Figure 7). However, several limitations should be noted. First, the initial transcriptomic screening in this study was based on only three paired bovine mammary tissue samples. Although this sample size is commonly used in exploratory RNA-seq studies, limited biological replication may still affect the robustness of the screening results [55]. Second, although the functional experiments in BMECs provided mechanistic support, the present study lacks in vivo functional validation in the bovine mammary gland, and therefore the biological role of BMNCR in the complex physiological environment of the lactating udder still requires further confirmation. Third, this study focused on *S. aureus*-induced bovine mastitis. Testing whether the BMNCR/miR-145/*ANO6* axis also operates in mastitis caused by other major pathogens (e.g., *Escherichia coli* and *Streptococcus*) would strengthen the generalizability of our findings and is an important direction for future work [24,56]. Overall, our findings provide a basis for further validation in bovine models and under additional pathogen backgrounds.

## 5. Conclusions

Our study demonstrates the involvement of BMNCR in *S. aureus*-induced bovine mastitis through systematic bioinformatics and experimental validation. BMNCR was significantly upregulated in *S. aureus*-associated mammary tissues and BMECs, and mechanistic analyses showed that it modulates *ANO6* expression by sponging miR-145. These findings indicate that the BMNCR/miR-145/*ANO6* axis contributes to inflammatory regulation and epithelial homeostasis during bovine mastitis. Overall, our findings suggest that BMNCR acts as a protective/compensatory regulator in *S. aureus*-induced bovine mastitis and may help limit excessive inflammatory injury while preserving epithelial homeostasis.

## Data Availability

The original contributions presented in this study are included in the article/Supplementary Material. Further inquiries can be directed to the corresponding authors.

## Supplementary Materials

The following supporting information can be downloaded at: https://www.mdpi.com/article/10.3390/ani16101446/s1, Supplementary Data: The full-length sequences of BMNCR were obtained by RACE; Table S1: qRT-PCR primer sequences of lncRNAs; Table S2: qRT-PCR primer sequences of miRNAs; Table S3: qRT-PCR primer sequences of genes; Figure S1: Establishment of a *S. aureus*-induced bovine mastitis model; Figure S2: Identification of primary bovine mammary epithelial cells; Figure S3: Coding Potential Prediction and Open Reading Frame Analysis of BMNCR; Figure S4: MiR-145 inhibition rescues the reduction in *ANO6* expression caused by BMNCR knockdown; Figure S5: Validation of the lncBMNCR/miR-145/*ANO6* regulatory axis in MAC-T cells.

## Abbreviations

BMNCR, bovine mastitis-related long non-coding RNA; S. aureus, Staphylococcus aureus; lncRNA, long non-coding RNA; ceRNA, competing endogenous RNA; miRNA, microRNA; BMECs, bovine mammary epithelial cells; MAC-T, bovine mammary alveolar cell-T; miR-145, bta-miR-145; *ANO6*, anoctamin 6; SCC, somatic cell count; LB, Luria–Bertani; qRT-PCR, quantitative reverse-transcription polymerase chain reaction; WT, wild type; MUT, mutant; EdU, 5-ethynyl-2′-deoxyuridine; CCK-8, Cell Counting Kit-8; ANOVA, analysis of variance; PCC, Pearson correlation coefficient; PCA, principal component analysis; DEG, differentially expressed gene; KEGG, Kyoto Encyclopedia of Genes and Genomes; GO, Gene Ontology; CPC, Coding Potential Calculator; ORF, open reading frame; MOI, multiplicity of infection.
