# Supplementary material for: LncRNA BMNCR Regulates Proliferation, Apoptosis and Inflammatory Response in Bovine Mammary Epithelial Cells Through the miR-145/ANO6 Axis
Source: Animals (Basel). 2026 May 8;16(10):1446. doi: 10.3390/ani16101446 (PMC13203851; doi:10.3390/ani16101446)
Supplement: Supplementary file 1 [file animals-16-01446-s001.zip › animals-4280135-supplementary.pdf]

Supplementary data: The full-length sequences of BMNCR were obtained by RACE

GAGTGTACCTGTAAGCAGTTGCATCCTTTCAAAAGCAAGCTCTTTGGATTCCTGGTTTCTGCTTTTCTAT
TTAAGAACGTAAAGAATTAAGGAGTATCATTAAATGGGCTTATGATGAATAATACATTATCTCAAGAATAA
TGCTTTAATTGCTGGAGACGCCATAAAGCTTGATAGTAATAATTTTATTTTAAAATGACAAAGATTTCTAC
ATCATGTTAATTTAGCCTTTGATACAGTAATAGAAACGGCTTTTAAAAAGATTCAAATACAGAAAAGCAT
TAAGGCGAGAGGTCTGGTTAAGCAGATGGGAGGTGATAGAACTGCAAATATTAGAATTGTGGTAACAT
TTGTATATACTAAAGATAAGAATACTGTTTCTGTATTAAAGTTGATAGGAAAAACTAGTTTTACATTTCTTT
TTCCCTCCTGCCAAATGAGAATATGGGATTTGGTGGCCGAAATTCCAGTTAGGTTTGAAATTCTTATTAA
GGACTTGGTTATTAGAGCTTTTGCATCTTCTTTGATCCAAAGTTATTTGCTTTTCAGCTGAAACTGTTTG
CTACTAGGTTGAACAAATAAATAAGCAGCATCTTGAATTCATTTTAGAACTTGGAATCCAGCATTTAAAG
GACACCAAGAGGGGCCTATTCATTGGGGTTTTATTTAGCTGGGCAGCAACTTCTCTTTCTGCCTGGTGA
AAAATGGATGTTTTCTCCTCGCTCAAACAACATAATCCTTTCTAATACAAAAGATTTAGACCTTGGAGTG
TGAAGGGTTAATGGTGGTCCTTCCCCGCTGTCCTCCCACCCCCCAGCTTCACCCCTGACTGTTTTGACA
ACACTTTTCAAAGTGTGACATTCCAAGCCCCTACATAACAATGTAATATTACTGTAATTACACAGGTCATT
ACATTTTAAATAGAGAACAATAAAATGCTTATCGCCCTGAAAAAGAACAGGTGTTCTTGCCCTTCTTTG
GTATTTATGCTAATTGTTTTTCATCTCCTTGAGAAATATTTATGGCAAACATGTTTAGATTTCAATCTCAAG
GCAGCAGTATTAATTCCGTGCTAATCTTTCTAGGTTGGGGTTTAATGTCTGCAGTTTGGATTATGGGCTC
AACATTCACCCAATAAGTCATATTTTAATGATTATAAATTTAATATGCCAGCGCTTCCACAGTTTGTTGAAA
TGAAAGCCTTGTGAAAACCCCAGAAACAAAACTATGAGGCATTCGGGAGCCTAGAGTGTTTATCTGGA
AGAAGTGTTTTTGGAAACTTGACTGCTGAAGGAAAGTAGCTTTCATTGATGCACCCCTATCTGATTGAG
AAAAGATTTGGAGAAAAACAATCCCCCATTCTGGCTGGGAGTTTTCCACCTTGATTTTAATTTTACTGAT
ACGGATTTCATTTAGAGTCCCTGCTGGAAACTGGTCAGCTTTCTGTTTCTTAGCAAGCTGTGAAAAAGT
TACAGGAAGGTTTCCGGGGGCTGGCAGATCAAGAATGCTGTTCTGTTAGCAGTTCAGGATAACTGCAG
TTCTGCACGCCATTAAAAAAATATTTGCTTTAGAAAACCAGCATTTTAATAGTTGCACCAGAGGCTGTGC
TTTTAAAGATTACAGTAGAAGTCTTTCCAGGTTTTGAATGTTTCCTCACATGCCACGAGGTGCCTGTGT
GCATACGAAAAGCACCCAGACTTCTTGGTGGAGAAATGTTGGTGCAACTTCTCTTACACAGGCTCAGT
GGGTAAACTGGAAGGTATGTAAGAGGATTTTACATTTTGTCAGATGTTTCACAACAGGGGCCCCCGAA
TCACATTAAAAATGCACTGCAGAGACAGGACAGCTGCAAATTTTTGTGCGGACGCGTGTTTCTGGACC
ATGACTAAAACTCCGGGGACCTTTTCCCCAGCTGTCTGGAAGTTCTGTGGAAGTTGGTTTATTGGCAGT
TAAGTCTGAGACAACCTGGATTTAGCAAAGACTCTCCTACACAGGAAGTAGATGACATTATATTGGGGG
AAGGGACTTCAGGCAAGGAGTGATTTACTCCTTTTTTGTTTTTTTTTTTAAACCGTGGCGTTGCCAAAT
AAATAAGCACTTTCCAAGGAAGTAGGTGTAAGCTTTTTAGTCTTGGTGAGTGGAAATAGATAATATCTTT
AACCGCATTTATAACTGTTTCGAGGTGACACATTTCAGAAGTTTGGAGAAGGAGGGGGGTGAATCAAA
TTCTTGAGGCTTTTCTTTTTGAAAGGGGTGTGTGATGTGGGTGTCTGAGTTTCTTCTGGGAGTGGATAT
TGGCAACCGTGTAAGTCAATCTTCAACTCTGACTTATAATTGAGCACATTTGTGAAGGAAATGGTTAATT
TTACAACCAAAGGCAAACTCGCACCTCGACAGGTCTGCTGAAAGAAATGTGCCCTAGTCCTTTGCTGG
CAAGCACCACCGGGTCATAAATCCACAGGCTTTATTTACAGCCCATAACACTTATGAATGTTAAGATATTT
GACGCCACGTTGGCCTTATAATATTCAAGGTCTGCAGACACTGGCAGTAGCTCAAATTTCTCTCACTAAT
TATAAGCAATGAGATGGGGGGGGGTGGAGACAGCCCATCGCCAAAGCTGCCCTCTCCCCCGCCTCTGA
CCCCCACCCTTACAATCTGAGCCAACCGAGACAGACACCCCCTCCTCCTCTGGCAGCTGACTTGTACTT
TTCCTCCAGGCTATTGTTATACACATTTATAAAGAGCACTTTCAGGAATGGAAATTCAGTGGGGGCTCTG
CACGAATGTTTTTCAAAAGCAAAGTTGCTTTAGGTGTTTGCATCTTTTTTCCTTTTCTTCTGGTTTATCCC
TTTTTCCTTTCTTTCTTGACTTTTTTTTTTTTTTTTGGAGACCCTGTTTTCTATATTGTGCTTATTTCCCAGA
ATACTTAAAGAAACCTAATATGGGAGGCTGCCCTTCAATTTACATGATGTTTCACTGAGCTGGAAGGGA

AAACGTACGCATTGCATAGTGCGCTAGCTAACGTGAGGGTTAAAAGAAAAGTGACGTGGAGTGAGGA
GAATTGATTAAAAGAATTAATAGATGAGGAAAAAAAAGCCAAGTTTGCTTTGGGAATGGGAAGGCCAT
TCATTTGGGAAATGTGCAGTGGTGTCTTGGTGGAGGACATAGAAATGTAACAGAGAAGGCAGAATAAA
TGCTTGGCTAAGAAAGGTTAGAAAGGCATAAAAAGATGCGGCAGAGATTTGGGCTGGACAAAAATGG
CTTCATTGAAATTAGGAGTTAGAAGTTTCTGATGAGAGGTGAAAATACCATGACTGGGGGCTGGGGTG
GGGGATGGTATTAACAACCATATTGATTTGCTCAGTAAAAACTGGTTCCAATCTAGTTTTCACCCAAAGA
GCTCTGTGCAGCAGATAATAATTTCATGTGAGTTACCGTGCAAGAGTCTGGGGAAGGGGCATCTGTGG
TGAAGACCCCCGATGTGAGAGAGATGGCCAGTTACCCTGTGGATTGTAATTTATTCTTAAAAAAGGAGT
GGGCTGTCCTGGTGTTTGTGTGACCACATGGCAAAGGGGGAGTGGGATTCCGAGGTGTGTGGGAAA
CTGTGGCAAAGAGGGGCGGGGCTGATGGCTGTGTCATTTCTGGCCGGGTCCTTCGCTCCATCCAACCC
AAACTTAAAGACTGAGGCTGAACGGTTTTATTTTCTAATCAATGGAATGCAGAAACTTACTAGCCCTCT
GGGACAGATAGGAATCCCTTAACAGCTCATTACAAAGATGGGGCTGTGGGATCTTTGAACGGGTTATC
AGCCCTGGTGTTCTTTGGTTTTCCTAGGATAGGCTGTTTTCTTCCTTAGGCATATCTGATTTGGGGCTGG
TAATTTGGTCTCTTATATTTTATCCTTTTTTTTTTTTGGTCATGAAGTAGCCCCACAACAACTCCTTCTGCA
GGGAAAAGGGATGGCTGTTAATGGGATCAGCTCTGAAGGAGTTAAAATGGCTTGCTAAAGTTTGGCAT
CATGAAAATAAATTTGAGGCCTGGTAGGCATTAGTAAGGCACAGCACATTTACAGAGCTTAATTAGTAC
GAACTGTAATTGTTCATGGTCTGCCAGAAGAAGTAATGTTCAGGAAAAGTGGAGTCCTTCTCTTGCAGT
CTTTAGTTTGAAACCGATAAATCACTTGCATATTTGTGTAGTGATTCAAATGGAGCTAAGACCTCTCCCAT
CCCAACATGTGGTAAATTGTAAAGTCAATGAATTGCAGATGTAGGCTACAGTGAGATTCTCTAAGAAAA
TGCAGAATGAATGGGAAAGAGTGATGCTATAAATATTTACTGAGAAGATAACTAGGTTCTTGCGTGCTG
GGCCAATTATGATGTACTAAGTATATTAGCAAAATGCTTAAAAAAAATTGAGAGGATTTGTATGGGTCTTT
GGGTGGGTTGGTTTATTTGGGAAAGTGAAGTAAAAGGCACTATGTTATTGCCCTGTCAGTTTTATTAAG
CCTGAATCACAATGGCATACCCAGGGAAGGACACATGAAACCCACATTAATGCAAATTAATTCGTTGTG
AGTTGCAGCACTGTGACTGCTAAGTGGAGTAATGATGGGGCATTATTTTAAGAGTGTTAGTGTAGCAAC
TTTTAATGAAAAATGCTGTGTTAGGAACATGTCTCAGCACTTTAGCCCACGTGTTTTTATATGCTGGAAA
TGTGTTTTGGACAAGAGCAGGAAACTTGCTTCACTTTATTCTTCTCTCTCCCAAACAGTTTAAGAACTGT
AATTATGGTTTGATGTTTAAAGAAAATCATCTAATTTTCCCTCAAAAAACAAATCCAGAAATGCCCATCA
CCATAACCAAGTTCAACTGTAACTTTTTGCAGTGACTGCACCACAAAATGGAAAAAAAAAAAATGTAGCT
CCCCCCACGCCCGCCATAAACCTATAGATATAACTTTCTCAAAGTGATTTTCTCTTTTTTTTTTTTTTTTTC
CTTAGAGAATTTTGGTCTCAGGCAGTGTTGCAAGTCAATACTTCTTTCCCTCAATGTGAGTAGGTGGAT
GTGTATGAAAGCTGTAAAAGTTAATGATCGTTCTCTGACTCATAGCTGATGGTTTCAGGTTGAGGAAAA
GAAAAGAAAAAATTAATGAGGGTAAATAGACATTTGATGGAACATCGAGACAGTAGGAGAGATTGTTC
CTGTGTCTAAAGGCGTGTGTTTTGTTGTGGCACAATTTCTGGAGAAAGCCAGGAAGGGAAAAGGGGT
TGCCTTTGTTAAGTTGTTGTTTTAGGAGGGTACAGATGTTCTGTGTCTGGATCTTTGCAGGAATGCTTAA
GCCTCCATCCCTACCAGTCTGTGCTGGTTTTGCCTTCCCTGTAGGAGTTAGAAGCCCCCTTGGCACTTTT
TAAAGGGGTAGGGAGACAGACACCCAGAGGCCTGGTCAGAAGTTTATTTCCCAGTAAATGTAAGAGG
CTCCCAACATAGGGCTTGGGGGTGCTGTCACTGTCCTGAAAAATGACTTGTGACCTAAGAAAAGGTCT
TAGATATACTTGGGCCCCTATTTTGTCTTTTATGCTAATGACATGTAAATTAAACTTAAAGTGCCTCTTGGT
GGTAGCTACAGTGGTGAGGAAGGTGAACCAAGAATTCCCCCAAACCAGAACCTTTTAGGAAATGAAT
CTGGATTTCTTCTTCTTTTTTTTTTAAATAGAAAGCTAGGTGATTCATAAAAATGGAAATAAAGCAAATG
CTCCCCCTCTTCTCAGTTTAGTGGAGTGAGTAGGCATACGAAATTAATCTTCATCCTACAGGAAGTTCAC
TTAAGTGTTGGAAAACCCAATGGAAGGTTTCAAGTTTTTTGAGTTGCCTTTGAGGATGAGATCTCTGTG
GCTGAAAAAGCTGCATTGCTTTTCTTCCATCTCTGGGTATCTCTGATGTCTTGTATCGGAAACATTGCATT
TA

Table S1. qRT-PCR primer sequences of lncRNAs

Items	Primers sequence（5’-3’）
CUFF.32343.2	F: AGTTCAGTCGCTCAGTTGTG<br>R: GAGGATGAGATGGTTGGATGG
CUFF.32737.1	F: AAGACTCTGATGCTGGGAAAG<br>R: CTTCCCTGTCCATCACTAACTC
CUFF.41043.1	F: TTAGTCGCTTCAGTTGTGTCC<br>R: AGGTCTTGGCAACTCATTCC
CUFF.43696.1	F: AATTCCATCACCTCCACTAGC<br>R: ATGACCCAGATAACCACGATG
CUFF.44897.1	F: TCAGTCATGTCCAGCTCTTTG<br>R: TGGGAAAGATTAAAGGCGGG
CUFF.44902.1	F: AGAAGGTGGAAAAGGAGCATC<br>R: AGAGCCGAGGAATAAATCACAG
CUFF.44994.1	F: GGAGTGTTTACAGTTGGAGAGG<br>R: GCATGGGTTTGATTTCTGGTC
CUFF.69486.1	F: CAGATTGCGTGAAATGTCCAG<br>R: CAACCCAAAAGAAATCCTGTCC
CUFF.71532.1	F: GGAAAGAGGTTGCAGTGAAAAG<br>R: GGGAAGCTAAGGAGAAAGTTGG
CUFF.56566.1	F: GGGAAAGAGCTAGAAGTTAGAAGG<br>R: AAGGAAAGTTGAGGTGATGGG

Table S2. qRT-PCR primer sequences of miRNAs

Items	Primers sequence（5’-3’）
miR-2284p	F: ACACTCCAGCTGGGTGAAAGTTTGTTC<br>RT: CTCAACTGGTGTCGTGGAGTCGGCAATTCAGTTGAGAAAATCCC
miR-126-5p	F: ACACTCCAGCTGGGCATTATTACTTTT<br>RT: CTCAACTGGTGTCGTGGAGTCGGCAATTCAGTTGAGCGCGTACC
miR-1185	F: ACACTCCAGCTGGGAGAGGATACCCTT<br>RT: CTCAACTGGTGTCGTGGAGTCGGCAATTCAGTTGAGAACATACA
miR-2441	F: ACACTCCAGCTGGGACAGGACAGGAC<br>RT: CTCAACTGGTGTCGTGGAGTCGGCAATTCAGTTGAGCTCCCACT
miR-877	F: ACACTCCAGCTGGGGTAGAGGAGATG<br>RT: CTCAACTGGTGTCGTGGAGTCGGCAATTCAGTTGAGCCCTGCGC
miR-1777b	F: ACACTCCAGCTGGGGGGGGCGGTGGG<br>RT: CTCAACTGGTGTCGTGGAGTCGGCAATTCAGTTGAGCCCCGCCC
miR-423-5p	F: ACACTCCAGCTGGG AAGCTCGGTCTGAGGC<br>RT: CTCAACTGGTGTCGTGGAGTCGGCAATTCAGTTGAGACTGAGGG
miR-2346	F: ACACTCCAGCTGGG ACTGATGTGAAGGT<br>RT: CTCAACTGGTGTCGTGGAGTCGGCAATTCAGTTGAGGCCAAACC
miR-455-5p	F: ACACTCCAGCTGGG GCAGTCCATGGGCA<br>RT: CTCAACTGGTGTCGTGGAGTCGGCAATTCAGTTGAGAGTGTATA
miR-451	F: ACACTCCAGCTGGG AAACCGTTACCATTA<br>RT: CTCAACTGGTGTCGTGGAGTCGGCAATTCAGTTGAGAAACTCAG
miR-145	F: GTCCAGTTTTCCCAGGAA<br>RT: CTCAACTGGTGTCGTGGAGTCGGCAATTCAGTTGAGAGGGATTC

Table S3. qRT-PCR primer sequences of genes

Items	Primers sequence（5’-3’）
GNB1	F: AATTAGAGATGCTCGGAAGGC<br>R: AGTGCATGGCATAGATCTTGG
ANK1	F: CAGAATATTCCAGGGGAGCA<br>R: CCTTGGAAATCACCTCCTCA
OSBPL1A	F: GGCGACTCTAGCCACTGAAC<br>R: GTCTCCACCACCACAGTCCT
ITGB8	F: ATTTTCCCTTTGAGCCCAGT<br>R: TGTGCATTGATGCTGAGACA
TMOD1	F: GGATGAGCTGGACCCTGATA<br>R: CTCGCTTCTCCCCTGTGTAG
ARG1	F: GGTGTGTGGGAAAAGCAAGT<br>R: GGAGTGTTGATGTCCGTGTG
DCUN1D5	F: ATGCCTTTGATTTTGCAAGG<br>R: AAGATCGGCATGGACTGTTC
CFL2	F: TGGGTATATGCCTCCTTTGC<br>R: TGAATGCTCAAGGCTCACAG
KPNA1	F: CTTCAGACCCGGATTGTGAT<br>R: TAGTCAGGCGGTTCTGCTTT
PSME3	F: ACTGAGGCTTCTGCCTACCA<br>R: GGAAGATCCCCTGAAGAAGG
CBFB	F: CCCGAAGAAGGACACGTGAA<br>R: GTCTTGTTGTCTTCTTGCCAGT
A1BG	F:AGCAGGCGACGTTCTTTGAC<br>RCTGAAACTCCTGGGTCGGC
DERL2	F: AGAAGGCTCTTTCCGAGGTC<br>R: GAGTTCCCCAACAACAAGGA
TNPO1	F: GGGGGAAGGGTAAACCAGTA<br>R: TGCTTCTTCACATCCAGTGC
RTN4	F: TTGGGGAATTGCAAAGAAAG<br>R: GGCAAGCTTTTTCACTCCTG
ACOT13	F: GGGGTGGGATAGGGAAATAA<br>R: GCACGAATCACCCCTAGGTA
SLC4A4	F: GGTGTGGACACTCCGAAACT<br>R: CCCAAAAGAGATCCAGGTGA
ST13	F: TGCTGGAGGAAAAGCAACTT<br>R: GGCAAACCGCTGTAAAATGT
PSAT1	F: CCGTGTTGCTAGAGATGCAA<br>R: GGACAGCACTGAACTGACCA
TAGLN2	F: CTTCCAGAACTGGCTCAAGG<br>R: ACACAGGCCATGTTCTTTCC
LOX	F: AGCTCAGCATACAGGGGAGA<br>R: CATCCATGCTGTGGTAATGC
ANO6	F: CTGGGTCATGAACGTGTTTG<br>R: AAGGTGACGAACCCAAACTG

IL-1α	F: GCTCAAAATGAAGACGAACCC<br>R: CAACTTTGGATGGGCAACTG
IL-2	F: GCACCTACTTCAAGCTCTACG<br>R: GGGCGCGTAAAAGTCAAATG
IL-6	F: AGAACGAGTATGAGGGAAAT<br>R: TGGCTGGAGTGGTTATTAG
IL-8	F: AAGAATTGAGAGTTATTGAGAGT<br>R: CAGACCTCGTTTCCATTG
IL-12	F: CACCAAAGATAAAACCAGCACAG<br>R: GGCACAGGGTTGTCATAAAAG
GAPDH	F: GGCAAGTTCAACGGCACA<br>R: ACCACATACTCAGCACCAGCA
β-Actin	F: TTCTAGGCGGACTGTTAG<br>R: TGCCAATCTCATCTCGTT
U6	F: CTCGCTTCGGCAGCACA<br>R: AACGCTTCACGAATTTGCGT
CXCR1	F: TGGTTGGTGACTCAGTCTTTC<br>R: CGTGCCGCTGTAATTTCCAA
BCL2	F: TCATGTGTGTGGAGAGCGTC<br>R:CTCCACAAAGGCGTCCCAG
SLC7A11	F: AAAACCATCCCCCTTGCGAT<br>R:CTCAGAAAAGGTCACCGCCA
GPX4	F:AATCCCAAGACCCGTGCG<br>R:CTCATTGCGAGGCCACATTG
PCNA	F:GTGAACCTCACCAGCATGTC<br>R:CAACGTGTCCGCGTTATCTT
ACSL4	F:GCCGACCTAAGGGAGTGATG<br>R:GTATCCTTTGGTCCCAGCCC

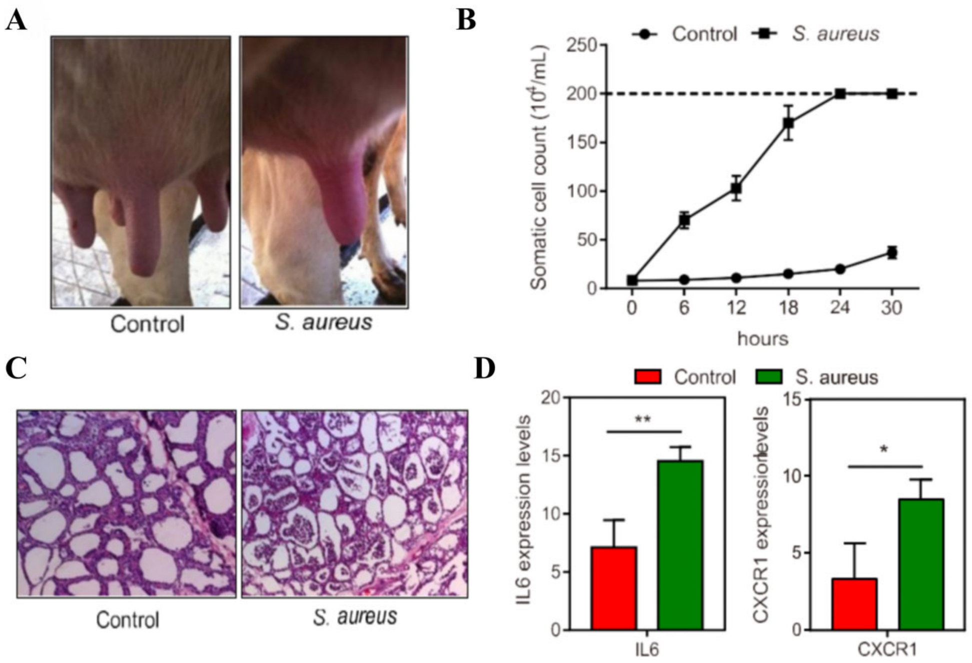

Fig. S1 Establishment of a *S. aureus*-induced bovine mastitis model.

After treatment with *S. aureus*. (A) Manifestations of redness, swelling, heat, and pain in bovine mammary tissue following *S. aureus* challenge. (B) Dramatic increase in somatic cell count (SCC) in milk. (C) H&E staining demonstrating exfoliation of mammary epithelial cells within mammary alveoli. (D) Significantly elevated expression levels of key inflammatory response genes (IL6, CXCR1). The data were shown as the mean ± SEM (n = 3) * $P < 0.05$, ** $P < 0.01$.

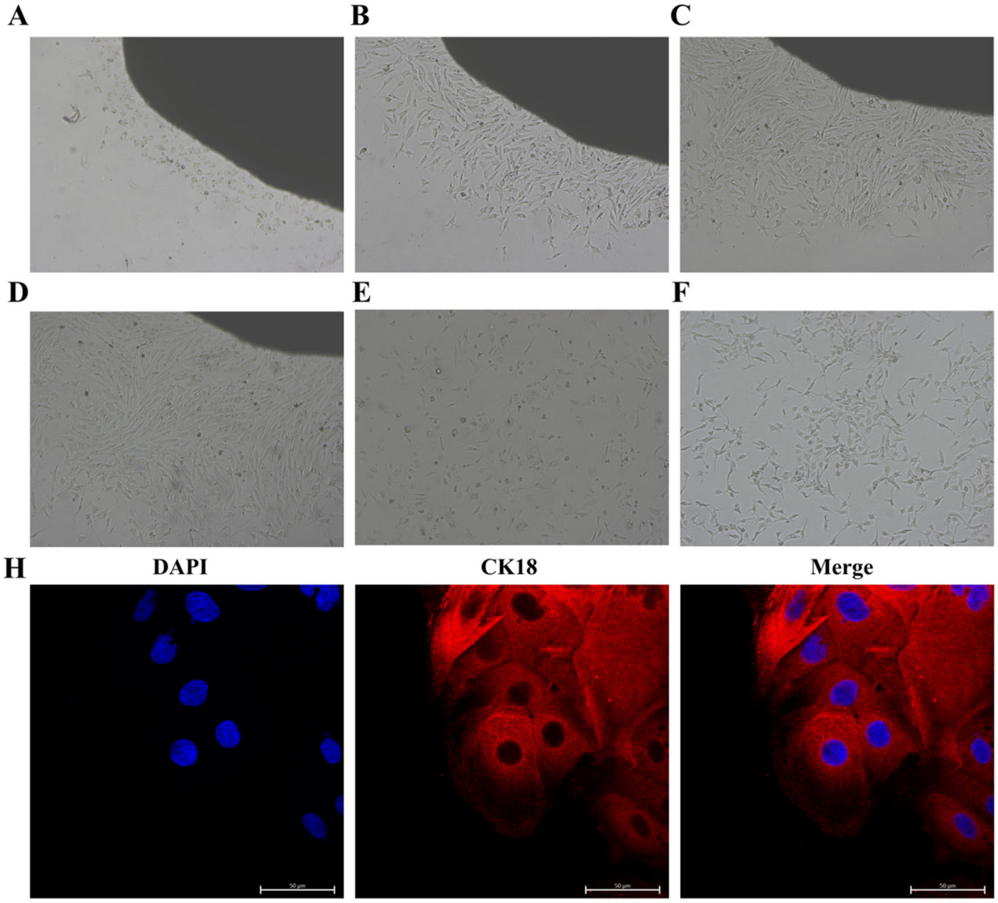

Fig. S2 Identification of primary bovine mammary epithelial cells

(A-D) Primary culture and outgrowth of mammary epithelial cells. (A) Day 1 of cell migration from tissue explants. (B) Day 2 of outgrowth. (C) Day 3 of outgrowth. (D) Day 4 of outgrowth. (E-F) Morphology of purified primary mammary epithelial cells: (E) cobblestone-like morphology; (F) mesh-like structure. (H) Immunofluorescence staining for CK18 (red) in primary bovine mammary epithelial cells. Nuclei were counterstained with DAPI (blue).

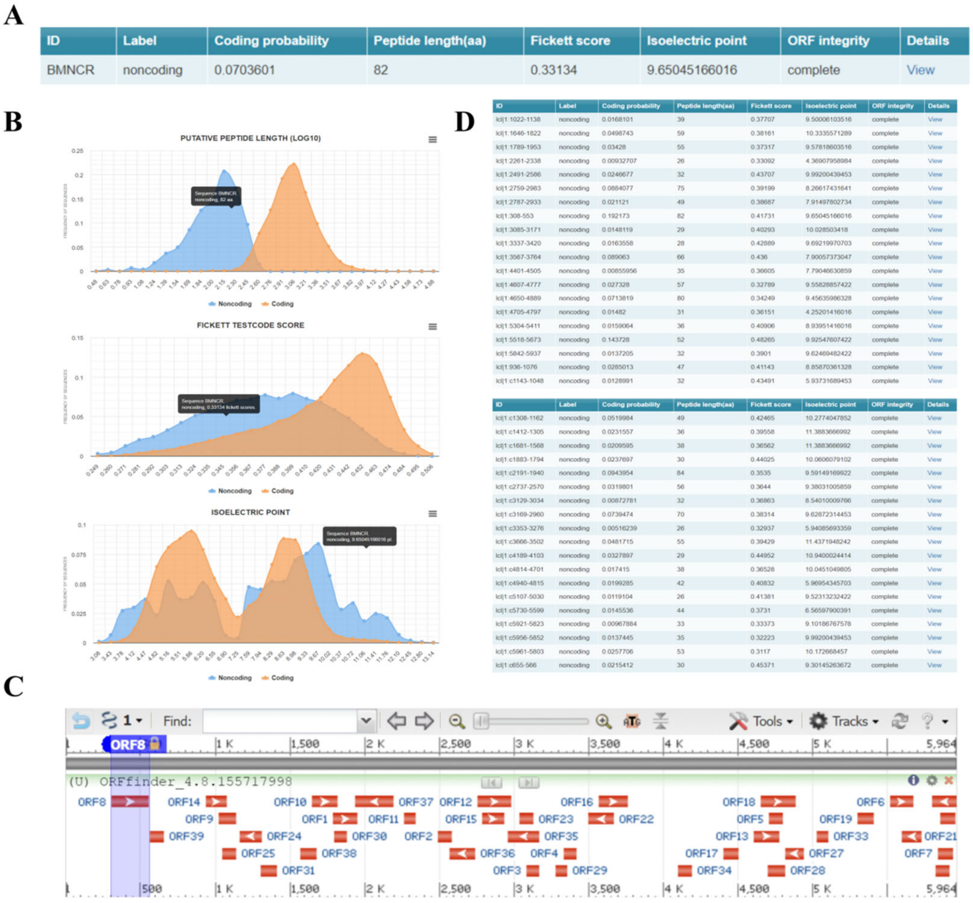

Fig S3. Coding Potential Prediction and Open Reading Frame Analysis of BMNCR

(A) Coding potential prediction of BMNCR. (B) Detailed characteristics of BMNCR including peptide length (aa), Fickett score, and isoelectric point. (C) Identification of 39 ORFs in BMNCR. (D) Functional assessment of the 39 ORFs.

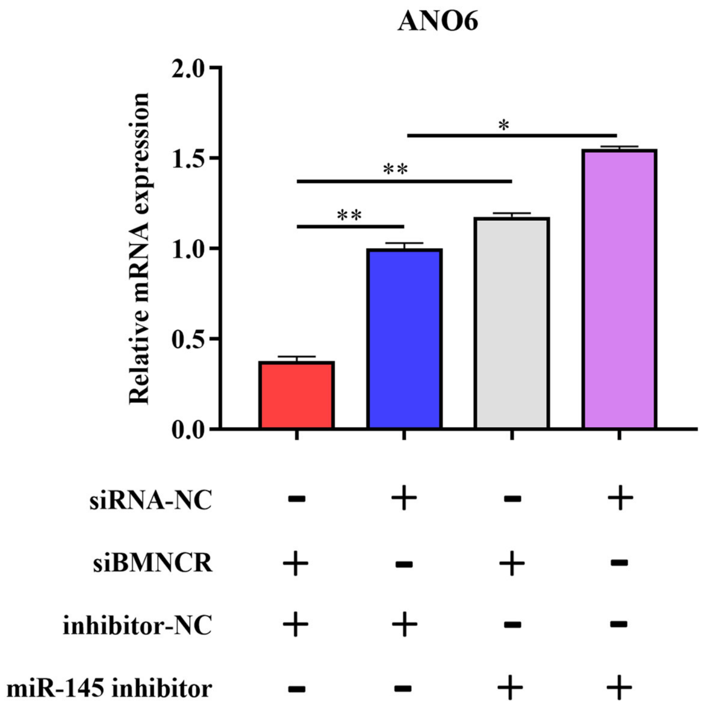

Fig. S4. MiR-145 inhibition rescues the reduction in ANO6 expression caused by BMNCR knockdown.

BMECs were co-transfected with the indicated siRNA and miRNA inhibitor, and ANO6 mRNA levels were assessed by qRT-PCR. The data were shown as the mean ± SEM (n = 3) * $P < 0.05$, ** $P < 0.01$.

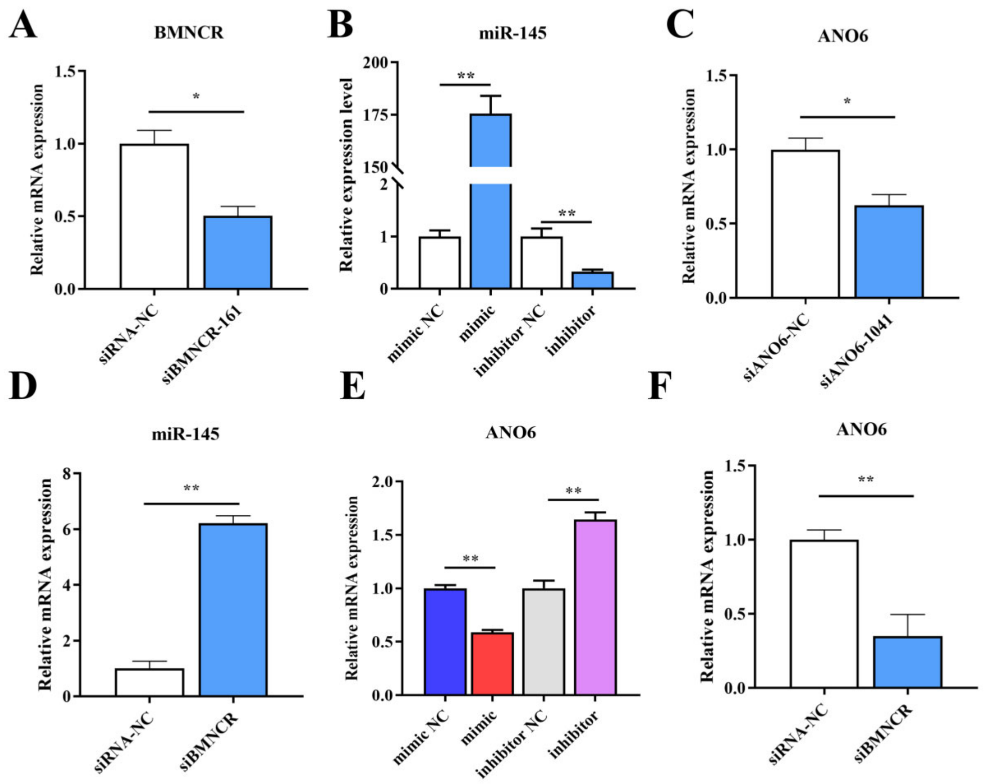

Fig. S5. Validation of the lncBMNCR/miR-145/ANO6 regulatory axis in MAC-T cells.

(A) Validation of BMNCR knockdown efficiency by qRT-PCR. (B) Validation of miR-145 overexpression or knockdown efficiency by qRT-PCR. (C) Validation of ANO6 knockdown efficiency by qRT-PCR. (D) Knockdown of lncBMNCR increased miR-145 expression. (E) ANO6 levels were increased by miR-145 knockdown and decreased by miR-145 overexpression. (F) lncBMNCR knockdown reduced ANO6 levels. The data were shown as the mean ± SEM (n = 3) * $P < 0.05$, ** $P < 0.01$.
